# Differentiating between renal medullary and clear cell renal carcinoma with a machine learning radiomics approach

**DOI:** 10.1093/oncolo/oyae337

**Published:** 2025-02-18

**Authors:** Rahim Jiwani, Koustav Pal, Iwan Paolucci, Bruno Odisio, Kristy Brock, Nizar M Tannir, Daniel D Shapiro, Pavlos Msaouel, Rahul A Sheth

**Affiliations:** Department of Interventional Radiology, The University of Texas MD Anderson Cancer Center, Houston, TX 77030, United States; Department of Interventional Radiology, The University of Texas MD Anderson Cancer Center, Houston, TX 77030, United States; Department of Interventional Radiology, The University of Texas MD Anderson Cancer Center, Houston, TX 77030, United States; Department of Interventional Radiology, The University of Texas MD Anderson Cancer Center, Houston, TX 77030, United States; Department of Imaging Physics, The University of Texas MD Anderson Cancer Center, Houston, TX 77030, United States; Department of Genitourinary Medical Oncology, The University of Texas MD Anderson Cancer Center, Houston, TX 77030, United States; Department of Urology, University of Wisconsin School of Medicine and Public Health, Madison, WI 77030, United States; Department of Genitourinary Medical Oncology, The University of Texas MD Anderson Cancer Center, Houston, TX 77030, United States; Department of Translational Molecular Pathology, The University of Texas MD Anderson Cancer Center, Houston, TX 77030, United States; David H. Koch Center for Applied Research of Genitourinary Cancers, The University of Texas MD Anderson Cancer Center, Houston, TX 77030, United States; Department of Interventional Radiology, The University of Texas MD Anderson Cancer Center, Houston, TX 77030, United States

**Keywords:** renal medullary carcinoma, clear cell renal carcinoma, radiomics

## Abstract

**Background:**

The objective of this study was to develop and validate a radiomics-based machine learning (ML) model to differentiate between renal medullary carcinoma (RMC) and clear cell renal carcinoma (ccRCC).

**Methods:**

This retrospective Institutional Review Board -approved study analyzed CT images and clinical data from patients with RMC (*n* = 87) and ccRCC (*n* = 93). Patients without contrast-enhanced CT scans obtained before nephrectomy were excluded. A standard volumetric software package (MIM 7.1.4, MIM Software Inc.) was used for contouring, after which 949 radiomics features were extracted with PyRadiomics 3.1.0. Radiomics analysis was then performed with RadAR for differential radiomics analysis. ML was then performed with extreme gradient boosting (XGBoost 2.0.3) to differentiate between RMC and ccRCC. Three separate ML models were created to differentiate between ccRCC and RMC. These models were based on clinical demographics, radiomics, and radiomics incorporating hemoglobin electrophoresis for sickle cell trait, respectively.

**Results:**

Performance metrics for the 3 developed ML models were as follows: demographic factors only (AUC = 0.777), calibrated radiomics (AUC = 0.915), and calibrated radiomics with sickle cell trait incorporated (AUC = 1.0). The top 4 ranked features from differential radiomic analysis, ranked by their importance, were run entropy (preprocessing filter = original, AUC = 0.67), dependence entropy (preprocessing filter = wavelet, AUC = 0.67), zone entropy (preprocessing filter = original, AUC = 0.67), and dependence entropy (preprocessing filter = original, AUC = 0.66).

**Conclusion:**

A radiomics-based machine learning model effectively differentiates between ccRCC and RMC. This tool can facilitate the radiologist’s ability to suspicion and decrease the misdiagnosis rate of RMC.

**Implications for practice**

Renal medullary carcinoma (RMC) is often misdiagnosed as clear cell renal cell carcinoma (ccRCC), leading to inappropriate treatment and disease progression. Machine learning-based radiomics models may accurately differentiate between ccRCC and RMC. A radiomics-based machine learning model outperformed a clinical data-based model, highlighting the potential value of providing information to the radiologist during diagnostic imaging to raise the suspicion of RMC.

## Introduction

Renal medullary carcinoma (RMC) is a rare, highly aggressive renal neoplasm accounting for less than 1% of all renal malignancies.^1,2^ It predominantly appears in young African-American men, with a direct association with sickle cell hemoglobinopathies.^1-4^ Considering its aggressive nature and its predilection for resistance against conventional modalities of therapy, the prognosis of patients with RMC can be poor.^5^ This is reflected in a mean overall survival of less than 12 months.^3,6,7^ In contrast, clear cell renal cell carcinoma (ccRCC) is the most prevalent subtype of renal cell carcinoma (RCC), comprising approximately 70%-75% of all renal malignant neoplasms.^8,9^ The prognosis of patients with ccRCC is substantially better than those with RMC.^10^

Though both RMC and ccRCC originate from the kidney, they are significantly different biologically. Clear cell RCC is hyper-vascular and typically arises from the proximal nephron and tubular epithelium, while RMC is hypo-vascular and arises from the distal nephron. On cross-sectional imaging, RMC presents as an infiltrative renal mass whereas ccRCC typically demonstrates an expansile growth pattern.^11^ However, diagnostic challenges arise because ccRCC can exhibit a range of presentations, with some infiltrative masses potentially being classified as ccRCC.^12^ Tanaka et al reported 133 cases of infiltrative renal masses, noting that 103 were RCC.^12^ Moreover, the treatment approaches for these two malignancies are vastly different.^13-15^

In the case of stages I-III ccRCC, initial treatment approaches are predicated upon nephrectomy.^9,16,17^ In the case of RMC, upfront platinum-based chemotherapy is the current consensus treatment, even in seemingly localized diseases with few exceptions.^18^ Upfront nephrectomy is recommended only for isolated primary tumors <4 cm, confined to the kidney. Unfortunately, a larger proportion of RMC patients undergo nephrectomy, leading to poorer survival outcomes.^12,19^

Further, the systemic therapies used for ccRCC, including vascular endothelial growth factor tyrosine kinase inhibitors, mTOR inhibitors, and immune checkpoint inhibitor therapies, are completely ineffective against RMC.^14,15,20,21^ Conversely, the cytotoxic and epidermal growth factor receptor targeted therapies used in RMC have no activity against ccRCC.^14,15,22,23^

This difference in treatment strategies highlights the importance of rapid and accurate diagnosis. The negative ramifications of incorrectly diagnosing RMC include inappropriate treatment and often rapid progression of the disease.^19^ Conventional morphologic and visual assessment approaches to differentiate RMC from ccRCC are based on computed tomography (CT) and magnetic resonance imaging.^24,25^ While biopsy could be used when kidney tumors are initially diagnosed on imaging, this is often not performed due to the assumption that the tumor is ccRCC. In this situation, a patient with RMC may be taken directly to surgery, an approach that delays the patient’s ability to receive cytotoxic chemotherapy, resulting in rapid metastatic progression. An imaging-based method to accurately determine tumor subtypes is important for initial RMC recognition. Radiomics is a data extraction strategy for medical imaging involving the mining of medical images for clinically relevant information, utilizing many specific imaging features that may or may not be visible to the human eye.^26^ In past studies that utilized radiomics-based strategies to differentiate between renal neoplasms, RMC was often excluded due to its rarity or low overall institutional sample size.^27^ There is an unmet clinical need to differentiate between the RMC and ccRCC to facilitate the accurate diagnosis and subsequent management of RMC. The objective of this study was to develop and validate a radiomics-based machine learning model to differentiate between RMC and ccRCC.

## Methods

### Study cohort

**Figure 1.**
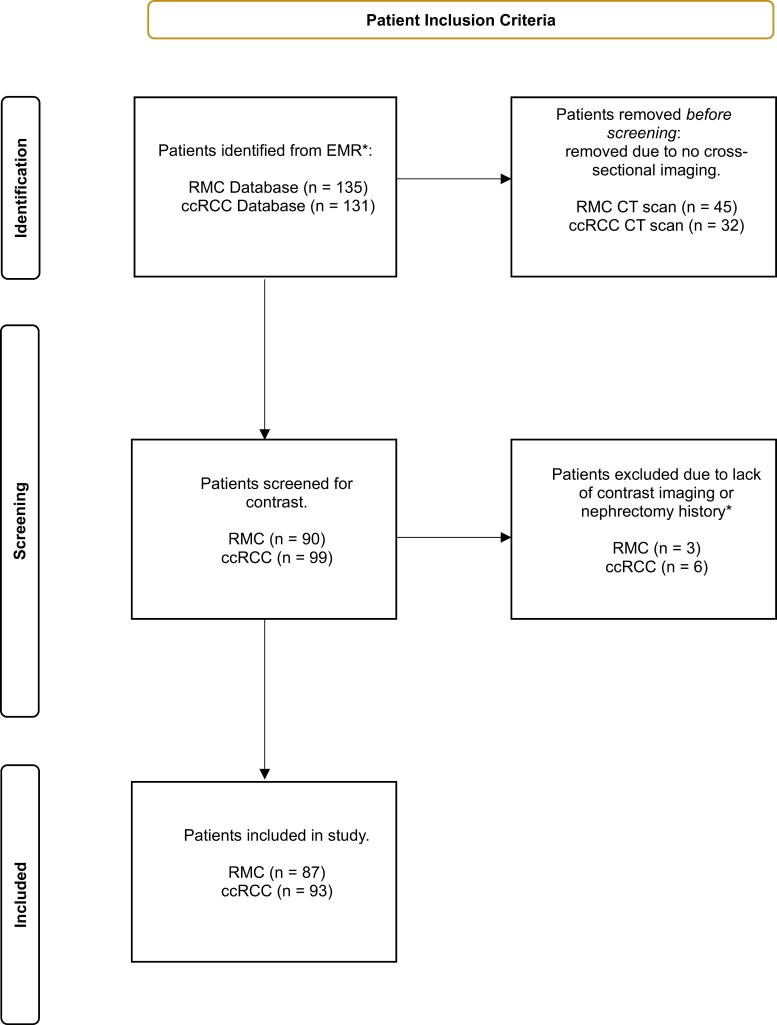
Patient inclusion and selection flowchart.

This retrospective study was approved by MD Anderson’s Institutional Review Board under protocols PA16-0736 and PA11-1045. An institutional database was queried to identify all patients diagnosed with RMC. A comparison cohort of patients with ccRCC was likewise identified from the institutional database. The initial study cohort comprised 135 (90 male, 45 female) patients diagnosed with RMC from July 2003 to November 2023, while the initial ccRCC list contained 131 (105 male, 26 female) patients diagnosed with ccRCC from 1998 to May 2023. Once patient populations were identified, they were screened for the availability and quality of computed tomography (CT) imaging studies. Patients without contrast-enhanced CT scans obtained prior to nephrectomy were excluded. Of the original list of 135 patients with RMC, 45 had no cross-sectional imaging available, as they had initially presented to an outside hospital and the imaging data were not available. Another removal of 3 additional patients from the cohort was due to a lack of contrast-enhanced CT imaging or a history of nephrectomy prior to CT imaging. Similarly, from the total of 131 patients identified in the ccRCC cohort, 32 were initially removed due to lack of available CT scans, followed by the removal of another 6 patients either due to the lack of contrast imaging or prior nephrectomy. With the inclusion criteria applied, the final RMC cohort comprised 87 patients, and the final ccRCC cohort comprised 93 patients (Figure 1). Overall, there were scans imported from 129 unique institutions and 9 different CT manufacturers (Supplementary Table S1).

### Image analysis and differential radiomic analysis

**Figure 2.**
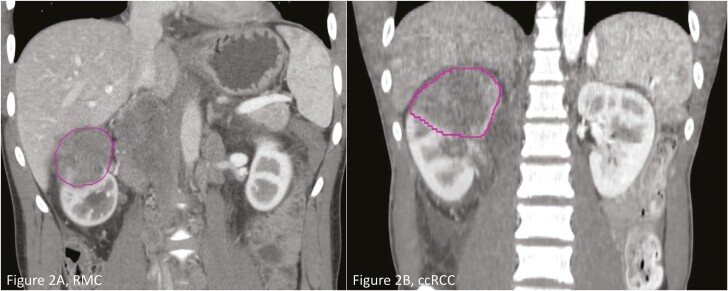
Segmented CT images. (A) RMC. (B) ccRCC.

CT scans were exported from the institution’s PACS server to a standard volumetric software package (MIM 7.1.4, MIM Software Inc.) for contouring (Figure 2). Contrast-enhanced CT scans were only analyzed for consistency, with the corticomedullary phase prioritized when available. For scans without available corticomedullary phases, the early venous phase was prioritized for segmentation.^28-30^ A board-certified radiologist (RAS) with 10 years of experience in volumetric image analysis reviewed the relevant contrast-enhanced CT scans for tumor features, including tumor size, shape, location, and invasion. Once identified, segmentation masks of the primary tumor were generated. Contouring was performed with both manual and semi-automatic tools, such as morphological contouring, to aid in creating accurate tumor masks.

Next, 3D quantitative radiomics features of the segmented tumors were extracted using PyRadiomics 3.1.0 after the application of the default preprocessing filters.^31^ The feature categories analyzed included first-order features, shape features (2D and 3D), grey level-co-occurrence matrix, grey level size zone matrix, grey level run length matrix, neighboring gray-tone difference matrix, and grey level dependence matrix features. In total, 949 imaging features were extracted per tumor segmented.

Radiomics preprocessing was performed with standard packages (RadAR) in the R environment (R Software version 4.2.3).^32^ Extracted texture features were standardized to remove each feature unit limit of data by data scaling with the min–max method. After scaling, data were normalized with quantile normalization, after which differential feature selection was performed with unsupervised clustering. We opted to use PyRadiomics and RadAR because of their open-source nature and established adoption within radiomics research.^31,32^ These tools offer comprehensive feature extraction and processing capabilities.

### Machine learning models

The study cohort was randomly divided and stratified into 70% and 30% subsets representing training and testing sets, respectively. Once prepared, the training sets were utilized for feature selection and for developing machine learning (ML) models via 5-fold-cross-validation. The overall prediction machine learning model was constructed using an extreme gradient boosting classifier (XGBoost). XGBoost was performed using R version 4.3.3.^33^ This study was conducted according to the Transparent Reporting of a Multivariable Prediction Model for Individual Prognosis Or Diagnosis (TRIPOD) guidelines.^34^

Several key strategies were employed to address overfitting. Feature selection was conducted using univariate methods and recursive feature elimination to focus the model on the most relevant features, reducing dimensionality. Regularization techniques, including L1 (Lasso) and L2 (Ridge), were applied within the XGBoost model, with hyperparameters optimized through grid search and cross-validation. Principal component analysis was also used to retain components explaining 95% of the variance. These measures combined to reduce model complexity, enhancing its generalizability and robustness.

### Parametric details

The model was trained with 100 boosting iterations (nrounds = 100), a learning rate of 0.01, and a maximum tree depth of 6. Class imbalance was addressed using a scale position weight ratio, with early stopping applied after 100 rounds. Regularization parameters included gamma = 1 to control tree complexity.

After utilizing the training data set on our ML model, the test data set was utilized to measure the model’s performance via the area under the receiver operating characteristic curve (AUC). As a secondary analysis, 2 additional ML models were developed for comparison. One model was used to investigate the potential impact of incorporating the presence of sickle cell trait into our predictive model, whereas the second model was used to investigate the ability to differentiate RMC and ccRCC based on commonly available demographic details (age, gender, laterality of the renal mass, ethnicity, and race) and no imaging data.

### Data calibration and stability analysis

Calibration metrics were calculated and plotted to assess the calibration of the developed ML model for overfitting. Calibration was then performed with a non-parametric approach using Bayesian binning into 7 quantiles.^35^ Given the relatively rare nature of RMC, stability metrics were then adopted, as suggested by Riley et al^36^ Bootstrapping was performed for 1000 iterations of the developed model to assess its prediction instability curve against bootstrapped models and the developed model and to assess the mean-absolute prediction error (MAPE).

### Statistical analysis

Statistical analysis was performed with R software version 4.3.3. A Wilcoxon test was used to compare the difference between training and testing set. Differential radiomics and relevant statistical testing were performed with the RadAR package.^32^ A comparison of radiomic features for ccRCC and RMC was performed using the Wilxocon-Mann-Whitney test. The receiver operating characteristic (ROC) curve was performed to determine the machine learning model’s performance in its ability to distinguish between RMC and ccRCC. The sensitivity, specificity, and area under the curve (AUC) were calculated. A *P*-value less than .05 indicated statistical significance.

## Results

### Patient demographics

Of the 87 patients within the RMC cohort, 31 were female, and 56 were male, with a median age of diagnosis of 29 (range 14-67 years) (Table 1). Similarly, of the 93 patients from the ccRCC cohort, 17 were female, and 76 were male, with a median age at diagnosis of 60 (range 35-78 years). These patients were randomly divided into training (*n* = 126) and test (*n* = 55) cohorts, whose characteristics are presented in Table 2. Scanner characteristics are available in Supplementary Table S1. Although 25 patients with RMC underwent upfront nephrectomy, only one of these patients was eligible for upfront nephrectomy per current consensus recommendations.^3^

**Table 1.** Demographic details.

Variable	ccRCC, *N* = 93^1^	RMC, *N* = 87^1^	*P*-value^2^
Age	60.00 (35.00-78.00)	29.00 (14.00-67.00)	<.001
Race			<.001
Black	6/93 (6.5%)	70/87 (80%)	
White	76/93 (82%)	9/87 (10%)	
Other	11/93 (12%)	8/87 (9.2%)	
Ethnicity			.028
Not Hispanic	75/93 (81%)	80/87 (92%)	
Hispanic	18/93 (19%)	7/87 (8.0%)	
Gender			.014
Male	75/93 (81%)	56/87 (64%)	
Female	18/93 (19%)	31/87 (36%)	
Sickle hemoglobinopathies			<.001
None	91/91 (100%)	7/86 (8.1%)	
Sickle cell trait	0/91 (0%)	76/86 (88.4%)	
Sickle cell disease	0/91 (0%)	1/86 (1.2%)	
Sickle cell beta thalassemia	0/91 (0%)	2/86 (2.3%)	
Unknown	0/91 (0%)	1	

^1^Median (range); *n*/*N* (%).

^2^Wilcoxon rank sum test; Pearson’s chi-squared test.

**Table 2.** Characteristics of test vs training dataset.

Characteristic	Training, *N* = 126^1^	Test, *N* = 55^1^	*P*-value^2^
Histology			.9
Clear cell	65/126 (52%)	29/55 (53%)	
Renal medullary carcinoma	61/126 (48%)	26/55 (47%)	
Age	46 (17)	45 (18)	.8
Race			.8
Black	54/126 (43%)	22/55 (40%)	
Caucasian	60/126 (48%)	26/55 (47%)	
Other	12/126 (9.5%)	7/55 (13%)	
Gender			>.9
Male	92/126 (73%)	40/55 (73%)	
Female	34/126 (27%)	15/55 (27%)	

^1^ *n*/*N* (%); mean (SD).

^2^Pearson’s chi-squared test; Wilcoxon rank sum test.

### Radiomics feature discrimination between RMC and ccRCC

**Figure 3.**
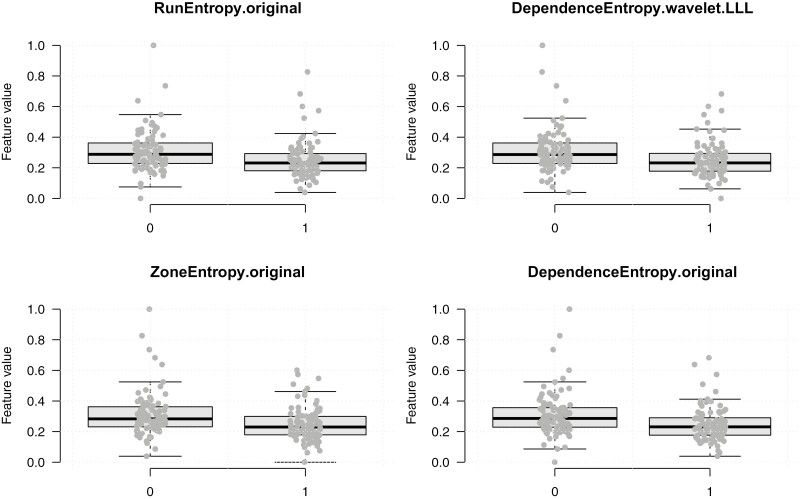
Boxplot demonstrating the 4 most important differential radiomic signatures. Multiple radiomics features within both the original CT images and pre-processed images were found to be significantly different between ccRCC (0) and RMC tumors (1). *P* < .01 for all variables.

A total of 949 imaging features across the filtered and original images, including shape, brightness, and texture, were analyzed to distinguish between RMC and ccRCC. Overall, the top 4 ranked features from differential radiomic analysis, ranked by their AUC were run entropy (preprocessing filter = original, AUC = 0.67), dependence entropy (preprocessing filter = wavelet, AUC = 0.67), zone entropy (preprocessing filter = original, AUC = 0.67), and dependence entropy (preprocessing filter = original, AUC = 0.66) (Figure 3). A correlation heatmap of the radiomic features is available in Supplementary Figure S1.

### Machine learning model generation and calibration

**Figure 4.**
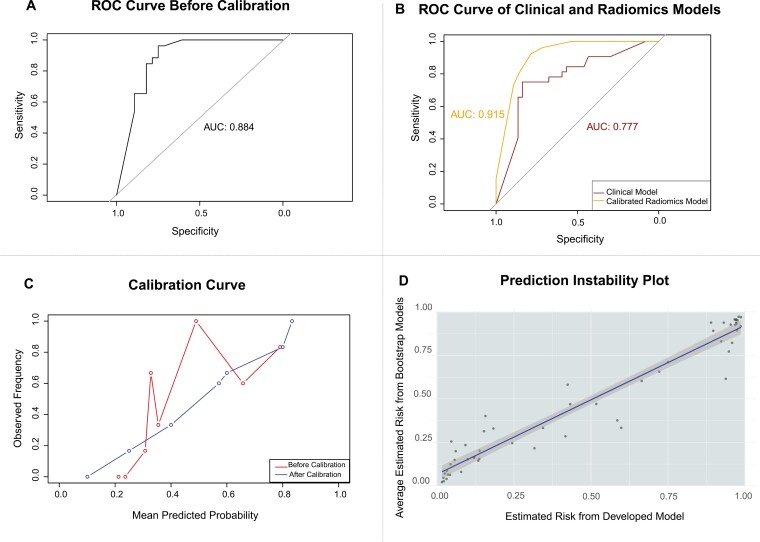
Receiver operating characteristic (ROC) curves of the ML models. (A). ROC curve of the uncalibrated machine learning XGBoost model (AUC = 0.884, sensitivity = 0.84, specificity = 0.83). (B) ROC curve demonstrating the difference in the clinical model (AUC = 0.777 *dark red, and lower curve*) with that of the calibrated radiomics model (AUC = 0.915 *orange, upper curve*). (C) Calibration curves were plotted before (blue) and after calibration (red) to assess the model’s overfitting. After performing Bayesian binning for calibration, the observed frequency is adjusted for a linear trend with the mean predicted probability and minimal overfitting. (D) Prediction instability plot. The slope of the original model (black) was 1.35 (95%CI 1.22-1.48). Grey denotes the 95% CI, with individual grey dots representing the average predictions of bootstrapped models.

**Figure 5.**
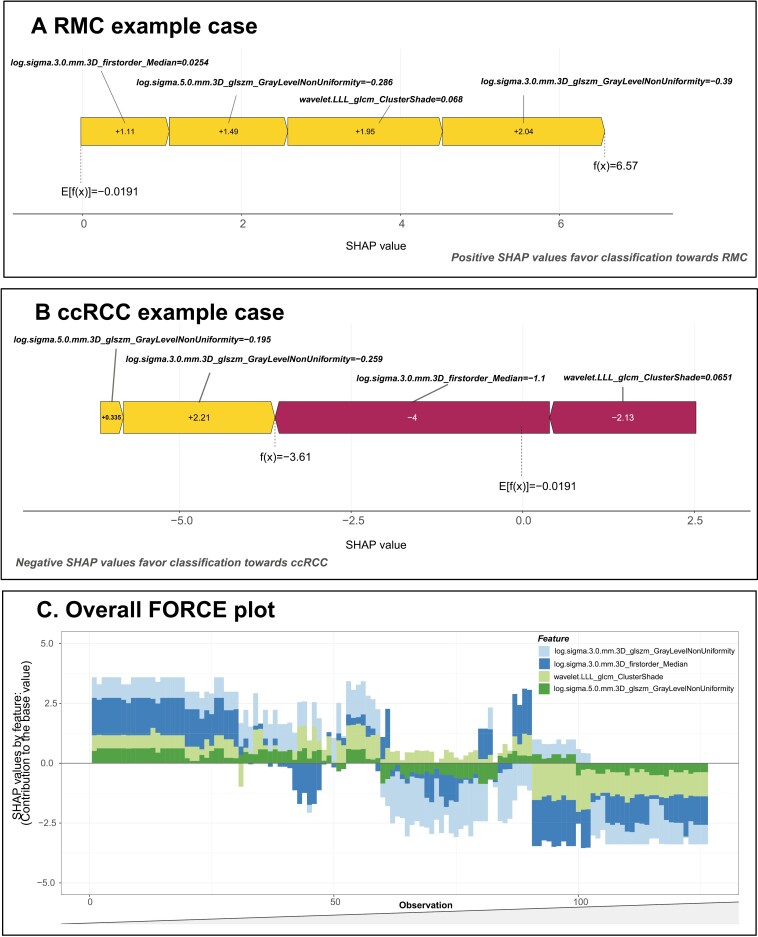
Shapley additive explanation (SHAP) force plots. The plots show the SHAP values for the first most important radiomic features used by the radiomics-based machine learning model. These radiomic features are log.sigma.5.0.mm.3D_glszm_GrayLevelNonUniformity (gain = 0.169), wavelet.LLL_glcm_ClusterShade (gain = 0.151), log.sigma.3.0.mm.3D_glszm_GrayLevelNonUniformity (gain = 0.144), and log.sigma.3.0.mm.3D_firstorder_Median (gain = 0.145). Overall, the trend in SHAP values is demonstrated over a range of feature values. (A) Example case of a RMC patient classified by the ML model. (B) Example case of a ccRCC patient classified by the ML model. (C) Overall force plot for the entire dataset. The x-axis is the observation number, whereas the y-axis represents the SHAP values. Positive SHAP values classify the observation toward RMC, whereas negative SHAP values classify the observation toward ccRCC. GLN measures the variability of gray-level intensity values in the image, with a lower value indicating more homogeneity in intensity values. Cluster shade is a measure of the skewness and uniformity of the GLCM. A higher cluster shade implies greater asymmetry about the mean. GLN measures the variability of gray-level intensity values in the image, with a lower value indicating more homogeneity in intensity values. First-order median represents the middle value of the intensity distribution in the ROI, providing a robust central measure of intensity that is less affected by outliers.

At a threshold of 0.5, the uncalibrated radiomics-based model demonstrated an AUC of 0.884, with a sensitivity of 84%, specificity of 82.8%, positive predictive value of 80.9%, negative predictive value of 85.7%, and F1 score of 82.4% (Figure 4). The calibrated model after Bayesian binning demonstrated an AUC of 0.92 (Figure 4B). The top 4 important features contributing to differential radiomic feature selection in the ML model were log.sigma.5.0.mm.3D_glszm_GrayLevelNonUniformity (gain = 0.169) and wavelet.LLL_glcm_ClusterShade (gain = 0.151), log.sigma.3.0.mm.3D_glszm_GrayLevelNonUniformity (0.144), and log.sigma.3.0.mm.3D_firstorder_Median (gain = 0.145) (Figure 5). Grey-level non-uniformity measures the variability of gray-level intensity values in the image, with a lower value indicating more homogeneity in intensity values. Cluster shade is a measure of the skewness and uniformity of the grey-level co-occurrence matrix. A higher cluster shade implies greater asymmetry about the mean. Grey-level non-uniformity measures the variability of gray-level intensity values in the image, with a lower value indicating more homogeneity in intensity values. First-order median represents the middle value of the intensity distribution in the ROI, providing a robust central measure of intensity that is less affected by outliers.^31,37^

Calibration curves were plotted before and after calibration to assess the model’s overfitting. Overall, there was a notable deviation from the ideal calibration line before calibration, particularly between bin ranges (0.318-0.332) and (0.415-0.591), signifying overfitting within these ranges of values. After performing Bayesian binning for calibration, the observed frequency was adjusted for a linear trend with the mean predicted probability and minimal overfitting (Figure 4C). The prediction instability curve of the developed ML model against the average of the 1000 bootstrapped iterations is shown in Figure 4D. The slope of the original model was 1.35 (95%CI 1.22-1.48). The MAPE for individual predictions within the developed model was 0.19.

### Secondary comparison models

A secondary ML model that incorporated sickle-cell trait with radiomics data was created. This model had an AUC of 1.0, driven almost entirely by the sickle cell trait characteristic (gain = 0.817) as the main determining feature differentiating RMC from ccRCC. A third model that utilized only clinical demographics such as age, gender, laterality of the renal mass on examination, ethnicity, and race was also created for comparison. This model was tested with the same parameters as the previously created models. This model prioritized age (gain 0.838) as the main determining feature to differentiate between RMC and ccRCC. The AUC was 0.78, the sensitivity was 97.6%, the specificity was 75%, F1 was 71.4%, the positive predictive value was 0.76, and the negative predictive value was 0.67. A comparative analysis of different machine learning classifiers (XGBoost, random forest, SVM, KNN, and logistic regression) is included in the Supplementary Figure S2.

## Discussion

This study demonstrates a robust differential radiomic analysis for differentiating between RMC and ccRCC exclusively based on radiomic features with a stable machine-learning model to effectively differentiate between the 2 histologies. RMC and ccRCC are biologically distinct renal neoplasms; differentiating between these malignancies is the critical first step in their appropriate management. While RMC typically arises in a specific patient population and thus could potentially be identified based on demographic characteristics, this malignancy is commonly misdiagnosed as ccRCC. Due to its rarity, a lack of awareness results in erroneous classification as a more common subtype of renal neoplasm. As a result, clinicians may erroneously opt for upfront nephrectomy in patients with RMC, even though consensus guidelines strongly recommend upfront systemic chemotherapy rather than nephrectomy for the vast majority of these cases with the exception of isolated primary tumors ≤4 cm in a greatest dimension that is confined to the kidney.^3^ Indeed, out of the 25 patients who underwent upfront nephrectomy in our RMC cohort, only one fulfilled the consensus criteria for this procedure. This highlights the importance of differentiating RMC from common tumors such as ccRCC, which are far more amenable to upfront nephrectomy. However, differentiating between the 2 histologies based on clinical imaging and reporting may be difficult alone. This was substantiated by our clinical ML model, which demonstrated an AUC of 0.78, compared to the calibrated radiomics mode, which demonstrated an AUC of 0.915.

Using differential radiomic analysis, the top 4 features that differentiated between renal medullary carcinoma (RMC) and clear cell renal carcinoma (ccRCC) were identified as 1 first-order feature (run entropy) and 3 second-order features (dependence entropy, zone entropy, and wavelet-transformed dependence entropy).^38^ While these features are not easily discernible to the human eye, they provide valuable insights into the texture patterns of the tumors. A higher run entropy indicates more irregular and complex texture patterns in RMC compared to ccRCC.

Regarding the second-order features, dependence entropy evaluates the entropy of gray-level dependencies in the native image, capturing the randomness, and variability in the texture. In contrast, wavelet-transformed dependence entropy emphasizes lower-frequency elements, allowing for the assessment of more coarse texture patterns, which can reveal broader structural differences between the 2 tumor types.^39^ The higher values observed for all 4 features in RMC compared to ccRCC reflect the greater heterogeneity and complexity of RMC textures (table 3).

**Table 3.** Top 10 differential radiomics features.

SI	Feature	Filter	Type of feature	Description	Statistic	Clear cell	RMC
1	RunEntropy	Original	first_order_statistics	Measures the uncertainty/randomness in the distribution of run lengths and gray levels. A higher value indicates more heterogeneity in the texture patterns.	0.667984	0.231585	0.288469
2	DependenceEntropy	wavelet.LLL	grey_level_size_zone_matrix	Measures the uncertainty/randomness in dependence.	0.665761	0.232246	0.286853
3	ZoneEntropy	Original	grey_level_size_zone_matrix	Measures the uncertainty/randomness in the distribution of zone sizes and gray levels. A higher value indicates more heterogeneneity in the texture patterns.	0.660573	0.229796	0.283073
4	DependenceEntropy	Original	grey_level_size_zone_matrix	Measures the uncertainty/randomness in dependence.	0.658226	0.230973	0.286113
5	LargeAreaHighGrayLevelEmphasis	wavelet.HHH	grey_level_size_zone_matrix	Measures the proportion in the image of the joint distribution of larger size zones with higher gray-level values.	0.653162	0.233508	0.292846
6	Imc2	Original	grey_level_co-occurrence_matrix	Informational Measure of Correlation 2 assesses the correlation between the probability distributions of *i* and *j* (quantifying the complexity of the texture). The range of IMC2 = [0, 1), with 0 representing the case of 2 independent distributions (no mutual information) and the maximum value representing the case of 2 fully dependent and uniform distributions	0.644516	0.234261	0.286876
7	LargeDependenceHighGrayLevelEmphasis	wavelet.HLL	grey_level_dependence_matrix	Measures the joint distribution of large dependence with higher gray-level values.	0.639081	0.224866	0.27866
8	LargeAreaHighGrayLevelEmphasis	wavelet.HLL	grey_level_size_zone_matrix	Measures the proportion in the image of the joint distribution of larger size zones with higher gray-level values.	0.638834	0.230369	0.286116
9	ZoneEntropy	wavelet.LLL	grey_level_size_zone_matrix	Measures the uncertainty/randomness in the distribution of zone sizes and gray levels. A higher value indicates more heterogeneneity in the texture patterns.	0.638587	0.240139	0.284555
10	GrayLevelNonUniformity	wavelet.HHL	grey_level_dependence_matrix	Measures the similarity of gray-level intensity values in the image, where a lower GLN value correlates with a greater similarity in intensity values.	0.636858	0.236796	0.284689

Furthermore, the developed ML model demonstrated durable performance metrics(AUC = 0.884, calibrated AUC = 0.92) to differentiate between the 2 histologies. Overall, this model can be a useful diagnostic tool that empowers the radiologist by highlighting potential RMC diagnoses, an important step in decreasing the inaccuracies in RMC diagnosis.

Like with all ML models, overfitting is a critical concern, especially for a rare and small cohort of patients such as RMC. Overfitting occurs when a developed model becomes excessively complex, capturing unwanted patterns, otherwise known as noise, within the training data as if they were a true signal, compromising the model’s ability to generalize to external test data sets.^40^ Ultimately, this results in the model lacking generalizability.^41^ One way to manage the problem of overfitting is to have a sufficiently large sample size and minimize the number of features extracted from the data set.^42^ Techniques performed to reduce the overfitting of this ML model included relevant feature selection, calibration with Bayesian binning into quantiles, quantile normalization, scaling, and 5-fold cross-validation. On the other hand, given the rarity of RMC, there may be an inherent level of instability within the predictions of the developed ML model.^36^ Model instability occurs because model prediction fundamentally depends on the sample and size of the data utilized. Even if the same method is used on a separate but similar data set, one could create a very different model with different individual predictions.^36,43^ To address this, bootstrapping with 1000 iterations was performed to assess the stability of the model predictions. Overall, the prediction instability demonstrated robust metrics with minimal variance (95%CI, 1.22-1.48) compared to the bootstrapped models. However, the MAPE was 0.189, which is only a moderate level of accuracy, underscoring the challenges when predicting outcomes for rare diseases and relatively sparse datasets such as RMC.

RMC is strongly associated with sickle cell trait. Not surprisingly, when sickle cell trait was included in the prediction model, the AUC was 1.0, with sickle cell trait being the most important feature. From a practical standpoint, for clinicians evaluating a patient with a renal mass and known sickle cell trait, the diagnosis of RMC can be readily achieved. In reality, however, this connection may not be made, and the diagnosis of RMC is missed because many patients are unaware of their sickle cell status.^3^ A tool that can raise the suspicion of RMC agnostic of sickle cell status is valuable to avoid a missed diagnosis. Thus, the pure imaging-based model is likely more clinically relevant than the one that includes the sickle cell trait. This tool can be used to screen patients with renal masses initially, and if the model raises a high likelihood of RMC, further evaluation with hemoglobin electrophoresis for sickle cell trait and renal mass biopsy can be recommended. Notably, a model that utilized commonly available demographic data performed inferiorly to the radiomics-based model, thus highlighting the substantial added value of imaging-based assessment for diagnosing RMC.

Previous studies that investigated renal masses and tumors from a radiomics perspective often excluded RMC due to its rarity in the population.^27^ Yu et al conducted a similar study in which they used a specific category of radiomics features, known as texture features, which is essentially a computerized interpretation of the subtle differences in pixel intensity to develop models to differentiate between RCC subtypes and oncocytoma.^27^ In their study, 3 models were created utilizing the support vector machine (SVM) method; the first model differentiated ccRCC from oncocytoma with an AUC of 0.93 and 0.91 depending on the texture feature kurtosis or skewness, respectively. The second model sorted oncocytoma from the papillary subtype of RCC with an AUC of 0.99 and 0.92 to differentiate oncocytoma from other tumor types. With the aid of ML, they also developed models to discern between ccRCC and other tumors with an AUC of 0.91 and a separate model that sorted between the papillary subtype of RCC and other tumors that displayed an AUC 0.92.^27^ The differential radiomics in our study was not limited to only texture features, although the features that most significantly were able to distinguish RMC from ccRCC were features from the texture category, RunEntropy, DependenceEntropy, ZoneEntropy, and DependenceEntropy.

Wang et al utilized radiomics to discern between ccRCC and non-ccRCC.^24^ They evaluated 3 models based on 4 features (Variance, HighGreyLevelRunEmphasis_AllDirection_offset7_SD, MinIntensity, and OneVoxelVolume) for which they utilized Artificial Intelligence Kit V3.0.0.R, GE Healthcare (AiK) for feature selection. For model construction, they utilized random forest (RF), SVM, and logistic regression (LR).^24^ In comparison to this study’s model, which had an AUC of 0.884 with a sensitivity of 0.84, and specificity of 0.83, their models performed similarly in terms of AUC, with their RF model (AUC 0. 909, sensitivity = 0.956, specificity = 0.538), their SVM model (AUC = 0.841, sensitivity = 1.0, specificity = 0.231), and LR model (AUC = 0.906, sensitivity = 0.956, specificity = 0.692).^24^ Interestingly, in addition to evaluating the performance of their models, they also compared them to that of a radiologist, who had a performance of AUC of 0.69, sensitivity of 0.850, and specificity of 0.581, demonstrating the potential utility of a radiomics-based screening tool.^24^

Machine learning models are often deemed as black boxes due to their complex mathematical formulae and difficulty in interpreting how the model is generated, especially for a physician.^44,45^ Overall, the developed ML model leveraged 4 distinct radiomics features to differentiate between RMC and ccRCC. Overall, RMC demonstrates a higher propensity to have more heterogenous, irregular, and variable radiomic signature when compared to ccRCC. Figures 5A and B are examples of a force plot that uses the Shapley Additive Explanations (SHAP) score to visualize how a model works and how positive or negative features drive the model toward classifying an observation into a particular model. Similarly to the work by Lundberg et al, our study not only demonstrates how the model individually classified observations but provides an overall explanation of how the model is applied to the entire dataset (Figure 5C).^46^ While it may not be completely necessary for clinicians to know how to code, the interpretability of how a model works and how particular features help leverage a particular observation into a final output should slowly gain traction to improve patient outcomes with the effective use of data science.

Limitations of this study include the retrospective nature of the study. As RMC is a rare cancer, there is a limited sample size, leading to an increased risk of type 1 error. Another limitation of this study is that the model constructed only differentiates between 2 types of cancer (ccRCC and RMC) and does not include other renal neoplasms that may be a part of the differential diagnosis. Furthermore, given the rarity of RMC, the availability of datasets for external validation to minimize the potential of overfitting is limited. Nevertheless, this study provides important insights into the radiomic characteristics of a rare under-investigated renal neoplasm and may serve as a foundation for future areas of study related to radiomics and RMC. Ultimately, this study demonstrates that radiomics combined with machine-based learning may be a powerful tool for differentiating RMC from ccRCC.

## Supplementary Material

oyae337_suppl_Supplementary_Tables_1_Figures_S1-S2

## Data Availability

Data cannot be shared for ethical/privacy reasons
