# Supplementary material for: Differentiating between renal medullary and clear cell renal carcinoma with a machine learning radiomics approach
Source: Oncologist. 2025 Feb 18;30(2):oyae337. doi: 10.1093/oncolo/oyae337 (PMC11833245; doi:10.1093/oncolo/oyae337)
Supplement: oyae337_suppl_Supplementary_Tables_1_Figures_S1-S2 [file oyae337_suppl_supplementary_tables_1_figures_s1-s2.docx]

Supplementary Table 1:

Scanner Characteristics

| **Characteristic** | **N = 185***^1^* |
| --- | --- |
| Manufacturer |  |
| Canon Medical Systems | 1 (0.5%) |
| GE MEDICAL SYSTEMS | 93 (51%) |
| Hitachi, Ltd. | 1 (0.5%) |
| Philips | 13 (7.1%) |
| SIEMENS | 62 (34.1%) |
| TOSHIBA | 13 (7.1%) |
| Unknown | 1 |
| Model |  |
| Aquilion | 10 (5.4%) |
| Aquilion ONE | 1 (0.5%) |
| Aquilion PRIME | 2 (1.1%) |
| Aquilion Prime SP | 1 (0.5%) |
| BrightSpeed | 3 (1.6%) |
| Brilliance 16 | 2 (1.1%) |
| Brilliance 64 | 5 (2.7%) |
| Brilliance Big Bore | 1 (0.5%) |
| Discovery CT750 HD | 25 (14%) |
| Discovery IQ | 1 (0.5%) |
| Discovery STE | 2 (1.1%) |
| Emotion | 1 (0.5%) |
| Emotion 16 | 1 (0.5%) |
| iCT 256 | 1 (0.5%) |
| Ingenuity CT | 3 (1.6%) |
| LightSpeed Pro 16 | 1 (0.5%) |
| LightSpeed Pro 32 | 2 (1.1%) |
| LightSpeed QX/i | 1 (0.5%) |
| LightSpeed Ultra | 2 (1.1%) |
| LightSpeed VCT | 14 (7.6%) |
| LightSpeed16 | 10 (5.4%) |
| Mx8000 IDT 16 | 1 (0.5%) |
| Optima CT520 Series | 1 (0.5%) |
| Optima CT540 | 1 (0.5%) |
| Optima CT660 | 7 (3.8%) |
| Revolution Ascend | 2 (1.1%) |
| Revolution CT | 4 (2.2%) |
| Revolution EVO | 4 (2.2%) |
| Revolution Frontier | 1 (0.5%) |
| Revolution GSI | 1 (0.5%) |
| Revolution HD | 13 (7.0%) |
| Sensation 16 | 4 (2.2%) |
| Sensation 64 | 6 (3.2%) |
| Sensation Cardiac 64 | 3 (1.6%) |
| Sensation Open | 1 (0.5%) |
| SOMATOM Definition | 2 (1.1%) |
| SOMATOM Definition AS | 11 (5.9%) |
| SOMATOM Definition AS+ | 4 (2.2%) |
| SOMATOM Definition Flash | 11 (5.9%) |
| SOMATOM Drive | 1 (0.5%) |
| SOMATOM Edge Plus | 3 (1.6%) |
| SOMATOM Force | 7 (3.8%) |
| SOMATOM go.All | 1 (0.5%) |
| SOMATOM go.Up | 3 (1.6%) |
| SOMATOM Perspective | 2 (1.1%) |
| SOMATOM Scope | 1 (0.5%) |
| Supria | 1 (0.5%) |
| KVP | 120.0 (90.0 - 140.0) |
| Slice Thickness | 3.0 (1.3 - 8.0) |
| Institution_Category |  |
| MD Anderson | 49 (26%) |
| Outside Institution | 136 (74%) |
| *^1^* n (%); Median (Range) |  |
|  |  |
|  | |

Supplemental Figures for RMC

**Supplemental Figure 1:**


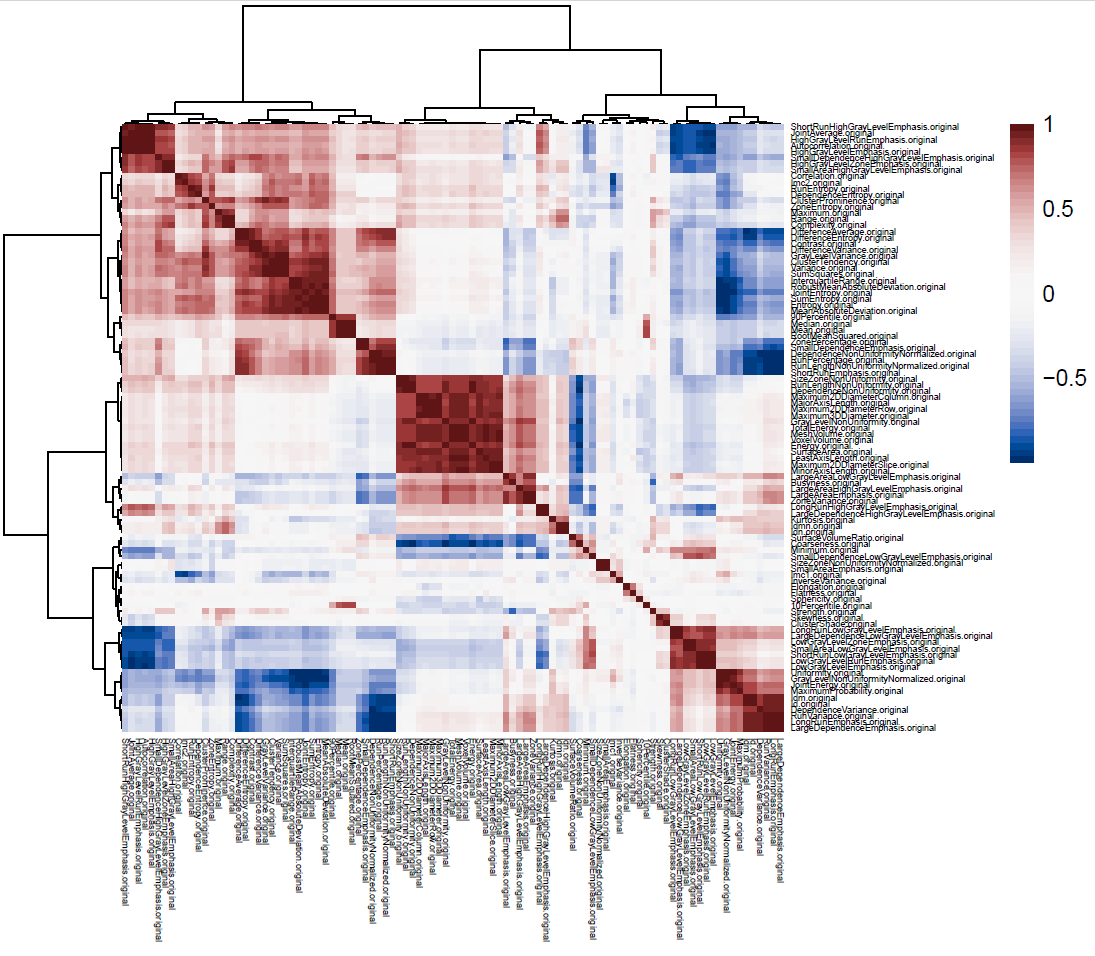


**Figure S1**. Correlation Heatmap of radiomic features, with the intensity of the color demonstrating the strength of correlation of a specific feature with another feature. The intensity of red demonstrates a positive correlation, while the intensity of blue demonstrates a negative correlation.

**Supplemental Figure 2:**


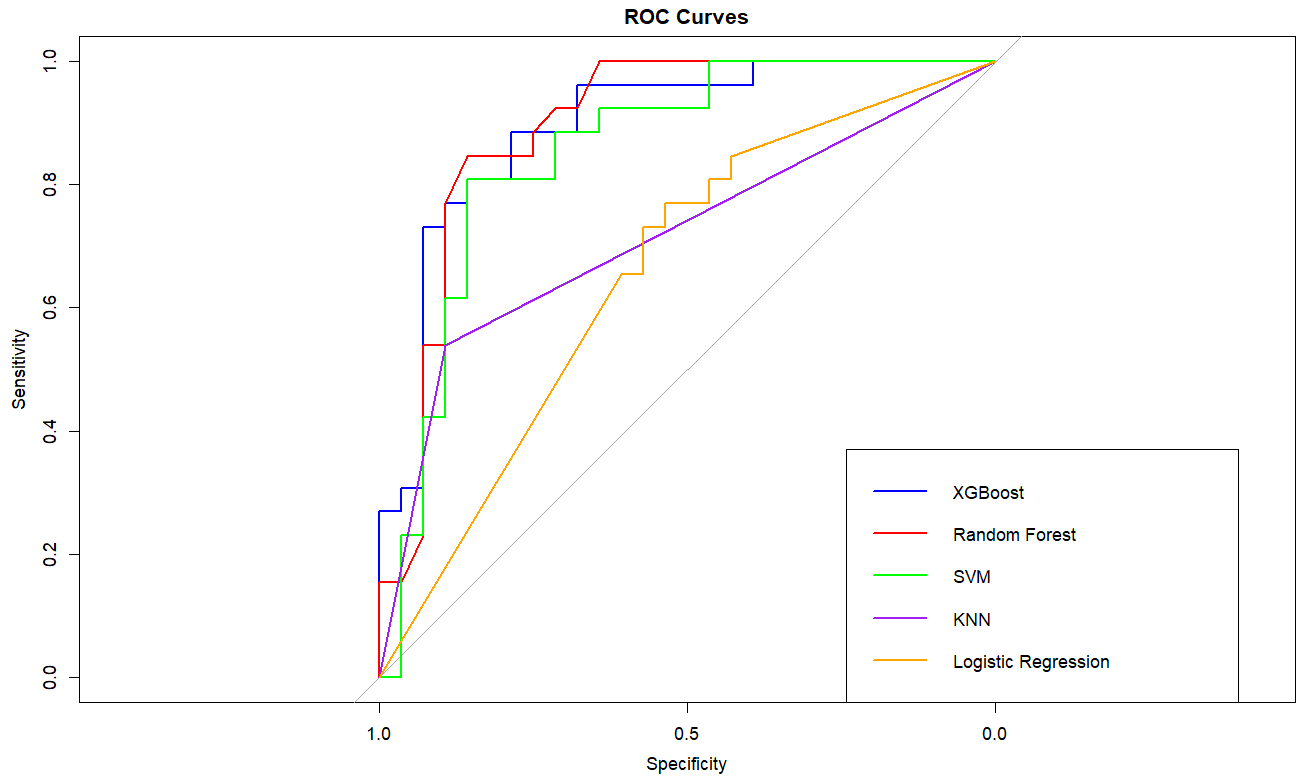


ROC Curves of the various models tested. The XGBoost model had a test error of 0.167 and an AUC of 0.894. The Random Forest model had a test error of 0.148 and an AUC of 0.894. The SVM model had a test error of 0.222 and an AUC of 0.853. The KNN model had a test error of 0.278 and an AUC of 0.716. The Logistic Regression model had a test error of 0.37 and an AUC of 0.657.
